# Complete Genome Sequences of Four Coxsackievirus A16 Strains Isolated from Four Children with Severe Hand, Foot, and Mouth Disease

**DOI:** 10.1128/genomeA.00760-17

**Published:** 2017-08-03

**Authors:** Shao-Jian Xu, Hong Yang, Xiang-Jie Yao, Hai-Long Zhang, Yan Ren, Wei Wu, Jun Meng, Hong-Biao Chen, Ya-Qing He, Ren-Li Zhang, Qi-Hui Lin, Long Chen

**Affiliations:** a District Key Laboratory for Infectious Disease Prevention and Control, Longhua District Center for Disease Control and Prevention, Shenzhen, People’s Republic of China; b Major Infectious Disease Control Key Laboratory and Shenzhen Public Service Platform of Pathogenic Microorganisms Repository, Shenzhen Center for Disease Control and Prevention, Shenzhen, People’s Republic of China; c School of Laboratory Medicine, Hubei University of Chinese Medicine, Wuhan, People’s Republic of China

## Abstract

Here, we report the complete genome sequences of four coxsackievirus A16 strains isolated from four children with severe hand, foot, and mouth disease. Three of them were assigned to subgenotype B1b based on phylogenetic analysis of the VP1 gene, and the other one belonged to subgenotype B1a.

## GENOME ANNOUNCEMENT

Coxsackievirus A16 (CV-A16), a member of the enterovirus A species of the family *Picornaviridae*, is one of the common pathogens of hand, foot, and mouth disease (HFMD) (1). Based on phylogenetic analysis of the VP1 gene, CV-A16 was classified into two main genogroups (A and B) and five genotypes (A, B1a to B1c, and B2) (2). Enteroviruses are genetically highly diverse and display considerable phenotypic variation (3). They can lead to a variety of clinical symptoms, ranging from mild HFMD, herpangina, upper and lower respiratory diseases, conjunctivitis, and gastroenteritis, to severe complications such as encephalitis, paralysis, myelitis, and meningitis. The molecular epidemiological studies of HFMD revealed that a majority of severe cases were caused by enterovirus A71 (EV-A71), whereas CV-A16 was often associated with mild HFMD (4, 5).

In 2014, seven CV-A16-positive severe cases from the sentinel surveillance system for HFMD were determined in Shenzhen, China. These CV-A16 strains were isolated by culturing clinical samples in rhabdomyosarcoma (RD) cell lines. Five CV-A16 strains, including a fatal strain, were selected to amplify the full-length genome as described previously (6). Amplified DNA products were sequenced by a commercial corporation (TaKaRa, Japan) using a primer-walking method. Genome-wide sequence analyses were performed using BioEdit version 7.2.5 and the program MEGA version 6.06 (7).

The four CV-A16 strains are 7,410 nucleotides (nt) in length, excluding the poly(A) tail. The 5′ untranslated (UTR) region contains 746 nt, followed by an open reading frame encoding the structural protein P1 (2,586 nt), the nonstructural proteins P2 (1,734 nt) and P3 (2,259 nt), and the 3′ UTR (82 nt). The contents of A, C, G, and U are 27.57 to 27.73%, 23.21 to 23.45%, 23.21 to 24.04%, and 24.80 to 25.25%, respectively, with G+C contents of 47.10 to 47.46%. Three of the four strains were assigned to subgenotype B1b based on phylogenetic analysis of the VP1 gene, and the other one belonged to subgenotype B1a. The complete nucleotide sequences (7,410 nt) and complete amino acid sequences (2,193 aa) of the genotype B1a strain (CVA16/Shenzhen36/CHN/2014) have variations at 726 to 748 sites (9.8 to 10.1%) and 35 to 42 sites (1.6 to 1.9%) compared to the three genotype B1b strains, respectively. The complete nucleotide sequences and complete amino acid sequences of the three genotype B1b strains have variations to each other at 237 to 259 sites (3.2 to 3.5%) and 15 to 22 sites (0.7 to 1.0%), respectively.

Phylogenetic analyses indicated that all of the CV-A16 strains determined in this study and the CV-A16 prototype strain G-10 (GenBank accession no. U05876) were monophyletic in the entire capsid protein P1 region. However, the four CV-A16 strains all segregated from G-10 and clustered with the other EV-A strains in the 5′ UTR and the nonstructural protein region (P2 and P3). This study has the same findings as previous ones, namely, that the prevalent CV-A16 of genotypes B1a and B1b in mainland China were potential recombinant viruses (8–10).

### Accession number(s).

The complete genome sequences of the four CV-A16 strains from this study have been deposited in GenBank under the accession numbers KX595291 to KX595294.
